# A facile bottom-up method for synthesis of peroxo-potassium titanate nanoribbons and visible light photocatalytic activity derived from a peroxo-titanium bond†

**DOI:** 10.1039/d2na00234e

**Published:** 2022-07-20

**Authors:** Hyunsu Park, Do Hyung Han, Tomoyo Goto, Sunghun Cho, Yukihiro Morimoto, Tohru Sekino

**Affiliations:** SANKEN (The Institute of Scientific and Industrial Research), Osaka University 8-1 Mihogaoka Ibaraki Osaka 567-0047 Japan hspark23@sanken.osaka-u.ac.jp sekino@sanken.osaka-u.ac.jp; Institute for Advanced Co-Creation Studies, Osaka University 1-1 Yamadaoka Suita Osaka 565-0871 Japan

## Abstract

Low-dimensional titanate nanostructures are gaining attention as a promising material for various photocatalytic applications. However, these conventional titanium oxide-based materials cannot utilize visible light because of their wide bandgap, and their synthesis generally requires high-alkali (10 mol L^−1^) and high-temperature (160–200 °C) conditions. Here, we report facile bottom-up synthesis for the visible light-activated peroxo-titanate nanoribbon (PTNR). The use of the peroxo-titanium complex ion containing the potassium ion as a precursor can induce the formation of a layered potassium titanate structure (K_2−*x*_H_*x*_Ti_2_O_5_) based on the self-organization reaction between titanium complex ions and potassium ions under mild synthetic conditions (0.29–4.39 mol L^−1^ KOH, 100 °C). Furthermore, the requirement of potassium ions in the formation of layered potassium titanate was stoichiometrically examined. The layered titanate crystals could be grown anisotropically, which depended on the radius of the cation used. Our results newly revealed that the larger radius of the interlayer cation promotes anisotropic crystal growth. As a result, in the case of the potassium base, a nanoribbon structure with a higher aspect ratio and larger specific surface area than those of lithium and sodium bases was formed. The formed peroxo-titanium functional groups significantly reduced the bandgap of titanate to 2.64 eV. In a photocatalytic decolorization test, the PTNR showed excellent photocatalytic performance based on the large surface area and enhanced light absorption in the visible light range while still performing well under UV light. These findings show not only that the proposed synthetic process has a low environmental impact but also that it contributes to the development of highly functionalized materials for photochemical applications.

## Abstract 2

A layered potassium titanate formed from the peroxo-titanium complex ions containing potassium ions is the basis for the formation of nanoribbon structures. This material exhibits excellent photocatalytic activity due to its reduced band gap.

## Graphical abstract

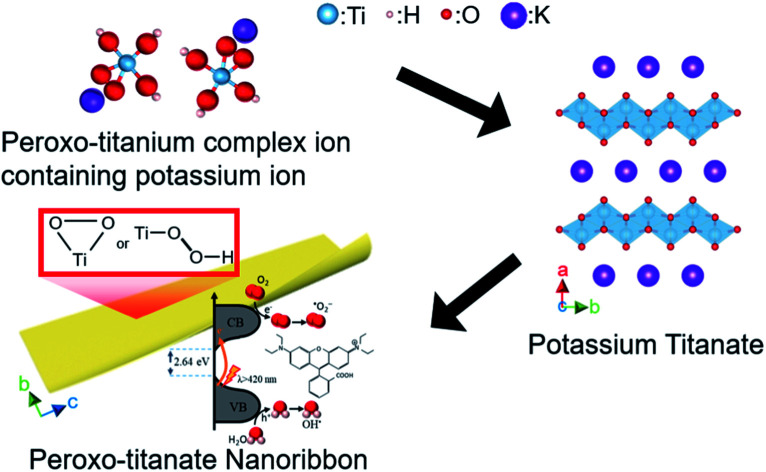

## Introduction

Contrary to commercial titanium dioxide (TiO_2_) polymorphs such as anatase, rutile, and brookite, layered alkali titanates have TiO_6_ octahedra that build up a layered structure in which the exchangeable alkali metal ion is situated in the interstitial space.^1^ Layered alkali titanates have the general formula A_2_Ti_*n*_O_2*n*+1_, in which A is a monovalent cation such as Na^+^, K^+^, or Li^+^.^2^ Researchers are interested in these unique layered crystal structures,^3^ which enable the formation of anisotropic low-dimensional nanostructures (*e.g.*, 1D or 2D). Recent global issues such as environmental pollution and viral infections have led to research on high-energy light-activated photocatalysts^4^ for their purifying,^5^ antibacterial,^6^ and antiviral^7^ effects. Several considerations for enhancing photocatalytic performance include the surface characteristics of the target substrate for adsorption and semiconducting properties for the photocatalytic reaction. This has increased interest in low-dimensional titanate nanostructures, which appear to be an optimal photocatalyst owing to their high specific surface area, remarkable redox and optical properties, and nontoxicity.^8^

Kasuga *et al.*^9^ were the first to report the synthesis of titanate nanotubes (TNTs) by simply treating TiO_2_ with NaOH. This simple method does not include any templates or surfactants and can also be used to synthesize other structures such as nanosheets,^10^ nanofibers,^11^ and nanowires.^12^ Furthermore, various alkaline raw materials such as LiOH,^13^ NH_4_OH,^14^ and KOH^15^ have been applied instead of NaOH to control the structure and morphology based on the strength of the alkali or the ion radius. Sikhwivhilu *et al.*^14^ reported that NH_4_OH cannot lead to the formation of layered titanate structures because the insufficient alkali strength leads to chemical changes in TiO_2_. Moreover, recently, layered structures such as two-dimensional structures have received great attention, and controlling the interlayer distance is an effective method of changing catalyst and ion storage characteristics.^16^ A larger interlayer distance can provide more accessible active sites for catalytic chemical reactions and ion capture.^17^ Therefore, many studies have been conducted based on the assumption that KOH is advantageous in synthesizing a layered alkali titanate having a wide interlayer structure because it is the strongest alkali and has the largest radius of cations among the materials mentioned above.^12,14,15,18,19^ However, most syntheses of layered titanates with KOH require an extremely high-alkali concentration and high temperature conditions as shown in Table S1 (ESI†). For instance, Sun *et al.*^20^ synthesized titanate nanobelts by treating TiO_2_ in 10 mol L^−1^ of KOH solution at 160 °C for 48 h. Du *et al.*^21^ reduced the process temperature of titanate nanowires to 130 °C but required a synthetic time of 3 days. Because these high synthetic temperatures cannot be reached at atmospheric pressure, hydrothermal synthesis using special containers such as an autoclave reactor with a Teflon chamber is required, in which the resulting product depends on the size of the synthetic system. Bavykin *et al.*^22^ tried to synthesize TNTs at a lower temperature of 56 °C, but this increased the synthetic time to 12 days. The use of an alkali is another factor that undermines the productivity and synthetic conditions (*i.e.* temperature or time). In experiments, 150 mL of 10 mol L^−1^ KOH solution was used per 2 g of TiO_2_ to synthesize layered potassium titanate (K_2_Ti_8_O_17_).^19^ Considering the theoretical yield and stoichiometric requirements of the K_2_Ti_8_O_17_ structure (*i.e.* molar ratio of Ti : K = 4 : 1), 0.35 g of KOH (0.006 mol) should be required for 2 g of TiO_2_ (0.025 mol) to obtain 2.29 g of K_2_Ti_8_O_17_. However, the KOH solution used in the experiment contained approximately 84.16 g of KOH, which is more than 240 times the stoichiometric requirement. Using KOH in large quantities can have a negative effect on cost and mass production. Furthermore, a strongly alkaline environment can cause the corrosion of experimental tools such as pipes or reactors and thereby reduce their lifespan.

The absorption and utilization of light are essential for photocatalysts. According to the band structure of semiconductors, light irradiation greater than the bandgap energy is required to excite electrons from the valence band to the conduction band during a photocatalytic reaction. Unfortunately, most titanium oxide-based materials including layered alkali titanates have high light reflectivity.^23^ These materials are visually white because they do not absorb and reflect visible light because they have a wide optical bandgap of more than 3.3 eV. These limitations allow the utilization of high-energy irradiation such as ultraviolet (UV) light to excite electrons for the photocatalytic reaction. However, the UV light region is less than 5% of the entire solar spectrum.^24^ In addition, although the use of photocatalysts has recently gained attention for use indoors as well as outdoors, artificial light such as that from lightbulbs and lamps does not contain UV light. Therefore, the focus of current photocatalyst research is extending the applicable spectrum to the range of visible light.^25,26^

Recently, our group reported the synthesis of a peroxo-titanium complex ion containing sodium ions and its use as a precursor to form a self-organizing layered sodium titanate structure.^27^ This bottom-up method of using an ion precursor allows the synthesis of layered titanate crystals at significantly lower alkali concentrations and temperatures, which results in the formation of anisotropic 1D nanotubes and enhances the visible light response by forming peroxo-titanium bonds (Ti–O–O).^4,28^ This technique has never been applied in the synthesis of layered potassium titanate; this is expected to be achieved simply by replacing the alkaline species in the ionic precursor. The versatility of our proposed method should be reviewed by dealing with the synthesis of similar materials to acquire the optimized technology of material property control. Therefore, the synthetic conditions for a complex ion precursor containing potassium ions needed to be established to facilitate the application of the precursor in synthesizing self-organizing layered potassium titanate.

In this study, we developed a low-alkali and low-temperature synthetic method for layered potassium titanate structures and clarified the effect of the inserted alkaline species on the crystal structure and morphology. Furthermore, the traditional precursors and synthesis methods are closely examined, and this emphasizes the superiority of our new methods. We investigated the presence and state of the peroxo-titanium bonds and the resulting visible light response. Finally, under light irradiation with a controlled wavelength, we evaluated the decolorization performance of Rhodamine B (RhB).

## Experimental

### Synthesis of the peroxo-titanium complex ion precursor

The peroxo-titanium complex ion precursor was synthesized by modifying the process in our previous study^27^ as follows. Initially, several solutions with a volume of 62.5 mL were prepared; only H_2_O_2_ (30%, FUJIFILM Wako Pure Chemical Corporation, Osaka, Japan) and mixed solutions with a pH adjusted to 6, 8, 10, and 12 by adding an aqueous solution of 10 mol L^−1^ KOH (85%, FUJIFILM Wako Pure Chemical Corporation, Osaka, Japan) to H_2_O_2_. All of solution preparation steps were carried out at 10 °C to prevent the increase in solution temperature due to the neutralization reaction. The pH was measured by using a pH meter (D-52, HORIBA Ltd., Kyoto, Japan). Then, 0.625 g of TiH_2_ (>99%, Kojundo Chemical Laboratory Co. Ltd., Saitama, Japan) was dissolved in the prepared solution, in which a vigorous exothermic reaction took place. Thus, a continual cooling process was needed. This was also carried out at 10 °C for 2 h.

### Synthesis of the titanate materials

The prepared ionic precursor solutions were heated in a refluxing vessel that was kept at 100 °C and stirred at 200 rpm for 24 h. Then, the precipitates were washed with distilled water by using a vacuum pump (MDA-020C, ULVAC Inc., Kanagawa, Japan) until the ion conductivity of the filtered solution became less than 5 μS cm^−1^. The precipitates were then dried by using a freeze dryer (EYELA FDU-2200, TOKYO RIKAKIKAI Co. Ltd., Tokyo, Japan). To evaluate and compare the structure, morphology, and optical and photocatalytic properties, traditional titanate compounds were synthesized in two ways. In solution chemical synthesis,^22,29^ 3 g of P-25 TiO_2_ (Degussa Evonik Corporation, Darmstadt, Germany) was added to 300 mL of 10 mol L^−1^ KOH aqueous solution (85%, FUJIFILM Wako Pure Chemical Corporation, Osaka, Japan), which was heated in a refluxing vessel at 113 °C for 24 h. Then, the precipitate was also washed and dried, and the prepared sample was labeled as “Titanate 1.” In the hydrothermal method,^19^ 1 g of anatase TiO_2_ (FUJIFILM Wako Pure Chemical Corporation, Osaka, Japan) was added to 80 mL of 10 mol L^−1^ KOH aqueous solution, which was moved into a Teflon-lined autoclave. This was heated at 160 °C for up to 4 days, and the resulting precipitates were washed and dried. The obtained samples were labeled as “Titanate 2” and “Titanate 3,” respectively. Finally, the pristine TNT^29^ was synthesized as a reference sample for comparing photocatalytic properties: 1 g of P-25 TiO_2_ was added to 400 mL of 10 mol L^−1^ NaOH aqueous solution (97%, FUJIFILM Wako Pure Chemical Corporation, Osaka, Japan), which was heated in the refluxing vessel at 115 °C for 24 h. Then, the precipitate was also washed and dried.

### Characterization

The crystal structure and purity data were examined by using X-ray diffraction (XRD) (D8 ADVANCE, Bruker AXS Co. Ltd., Karlsruhe, Germany). The diffraction patterns of structures were collected by using a Scintag diffractometer operating in the Bragg configuration and using Cu Kα radiation (*λ* = 1.54 Å) in the range of 5°–80° at a scanning rate of 0.02°. X-ray fluorescence (XRF) (ZSX100e, RIGAKU, Tokyo, Japan) was used to collect chemical analysis data, which were used to calculate the K/Ti ratios of each sample. The morphology and particle size of the samples were observed by using field-emission scanning electron microscopy (FE-SEM) (SU-9000, Hitachi High-Technologies Corporation, Tokyo, Japan) at an acceleration voltage of 30 kV. High-resolution transmission electron microscopy (HRTEM) (JEM-ARM200F, JEOL Ltd., Tokyo, Japan) was performed at an acceleration voltage of 200 kV to further characterize the individual nanostructures of the titanate. N_2_ adsorption–desorption experiments were performed by using a gas sorption analyzer (NOVA 4200e, Quantachrome Instruments, USA) to obtain the pore size distribution of the samples. The specific surface areas were calculated from the adsorption isotherm by using the Brunauer–Emmett–Teller (BET) method. Fourier transform infrared spectroscopy (FT-IR) (FT/IR4100, JASCO, Tokyo, Japan) was used in the transmission mode to obtain spectra in the wavenumber region of 4000–500 cm^−1^ at a resolution of 4 cm^−1^. The samples were sandwiched in a KBr plate and pressed to obtain pellets. X-ray photoelectron spectroscopy (XPS) was performed to measure the O_1s_ and Ti_2p_ spectra. The binding energy was standardized with C_1s_ energy. An ultraviolet-visible (UV-vis) spectrophotometer (V-650, JASCO Co., Tokyo, Japan) was used in the solid sample measurement mode to evaluate the absorbance of the samples in the wavelength region of 250–800 nm and for diffuse reflectance spectroscopy (DRS) with the Tauc plot method and Kubelka–Munk transformation to measure the optical bandgap energy. Another spectrophotometer (UVmini-1240, SHIMADZU Corporation, Tokyo, Japan) was used to record the absorption spectra of the decolorization testing solution.

### Decolorization test

The photocatalytic properties of the prepared photocatalysts were evaluated in a decolorization test, where RhB was used as an organic compound. The RhB solution was prepared with a concentration of 10 mg L^−1^. The test was carried out in the aqueous phase in a stirred batch reactor containing 2 g L^−1^ of photocatalyst suspended in 50 mL of the substrate solution. The solution containing the photocatalyst was stirred at 300 rpm in the dark for 30 min to achieve adsorption–desorption equilibrium of the organic molecules on the catalyst surface. Then, the solution was irradiated with controlled light, three types of light were prepared. First, UV light was irradiated with an UV irradiator (TOSCURE 100, Toshiba Lighting & Technology. Co. Ltd., Japan). The wavelength was limited to 280–380 nm by using a U340 bandpass filter (Hoya Corporation, Tokyo, Japan). Second, visible light from a solar simulator (OTENTO-SUN III, Bunkoh-Keiki Co. Ltd., Tokyo, Japan) was calibrated under a standard air mass to 1.5 and 1000 W m^−2^. Wavelengths below 420 nm were eliminated by using an L-42 bandpass filter (<420 ± 5 nm, Hoya Corporation, Tokyo, Japan). Third, solar light was irradiated with the solar simulator without any bandpass filter. Fig. S1 (ESI†) shows the light spectra. The samples were obtained at reaction time intervals of 1 h and were filtered by using a filter with 0.2 μm-size pores to remove the catalysts. Then, their absorption spectra were recorded. The decolorization of RhB was calculated according to the formula^30^ of decolorization = *C*/*C*_0_, where *C* and *C*_0_ are the absorbances of the RhB after the reaction and the initial RhB, respectively. The photocatalytic decolorization test was conducted three times to confirm the reproducibility, and each error bar was expressed.

## Results and discussion

### Structural features of titanates synthesized by the bottom-up method

**Fig. 1:**
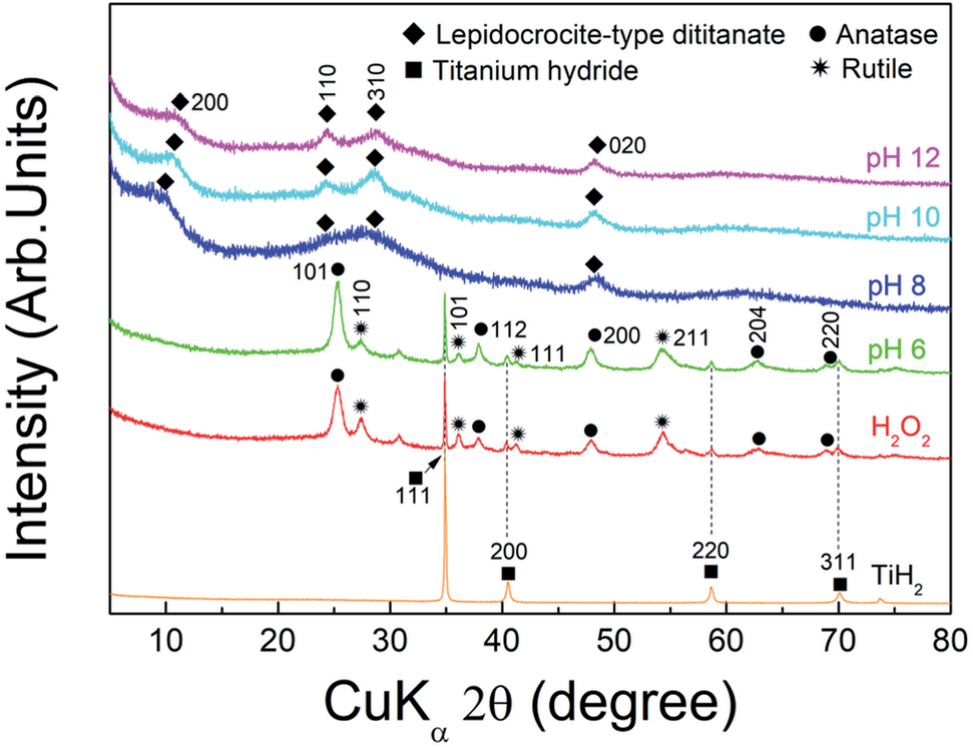
XRD patterns of powders synthesized from the ionic solution at the conditions of H_2_O_2_, pH 6, 8, 10, and 12, respectively, and commercial TiH_2_ powder.

To determine the optimal pH and amount of potassium ions for synthesis of the complex ion precursor, TiH_2_ was chemically treated under various pH conditions. Then, the prepared ionic solutions were directly applied as precursors for solution chemical synthesis. Fig. 1 shows the XRD patterns of the samples prepared from the various ion precursors and commercial TiH_2_ powder as the starting material. The pattern of TiH_2_ (PDF card# 00-003-0859) with a cubic structure and a slightly lower diffraction intensity was observed at the samples labeled as H_2_O_2_ and pH 6, respectively, and patterns assigned to anatase-TiO_2_ (PDF card# 00-021-1272) with a tetragonal structure and rutile-TiO_2_ (PDF card# 00-021-1276) with a tetragonal structure are also detected. While the sample prepared at pH 8 showed relatively low crystallinity, patterns assigned to lepidocrocite-type dititanate (PDF card# 00-047-0124) with an orthorhombic structure were detected above pH 8. TiH_2_ can be dissolved in the presence of H_2_O_2_,^31^ and this reaction is promoted with increasing pH.^32^ Similarly, our previous report^27^ showed that the ionization of TiH_2_ is promoted by increasing the pH of the H_2_O_2_ and NaOH mixed solution, which results in the formation of the peroxo-titanium complex ion with a transparent yellow color (assigned to [Ti(OH)_3_O_2_]^−^).^33,34^ Therefore, the results indicate that TiH_2_ was ionized into the peroxo-titanium complex ion in the H_2_O_2_/KOH mixed solution when the pH was above 8 according to the following reaction:

1 TiH_2_ + 3H_2_O_2_ + KOH → Ti[(OH)_3_O_2_]^−^ + 2H_2_O + K^+^ + H_2_

The prepared peroxo-titanium complex ion finally formed into lepidocrocite-type titanate (K_2−*x*_H_*x*_Ti_2_O_5_) by interaction with potassium ions according to the following reaction:

2 2[Ti(OH)_3_O_2_]^−^ + (2 − *x*)K^+^ + *y*H^+^ → (K_2−*x*_·H_*x*_)Ti_2_O_5_↓ + 3H_2_O + O_2_

The dissolution of TiH_2_ is given by^35^

3 TiH_2_ + H_2_O_2_ → TiO_2_^2−^ + 2H^+^ + H_2_

However, our results indicated that H_2_O_2_ and pH 6 are inappropriate conditions for this reaction because they do not provide the amount of potassium needed to form potassium titanate. Thus, more stable structures (*e.g.*, TiO_2_) form:

4 TiO_2_^2−^ → TiO_2_↓

The angles at the 200 plane of lepidocrocite-type titanate, which indicates the lattice interlayer distance,^36^ were 8.7 Å (10.18°), 8.4 Å (10.47°), and 8.2 Å (10.77°) at pH 8, 10, and 12, respectively. The differences in the structural properties may depend on the alkali inserted between layers, which may be related to factors such as the alkali type, binding properties, and amount of inserted alkali.

**Fig. 2:**
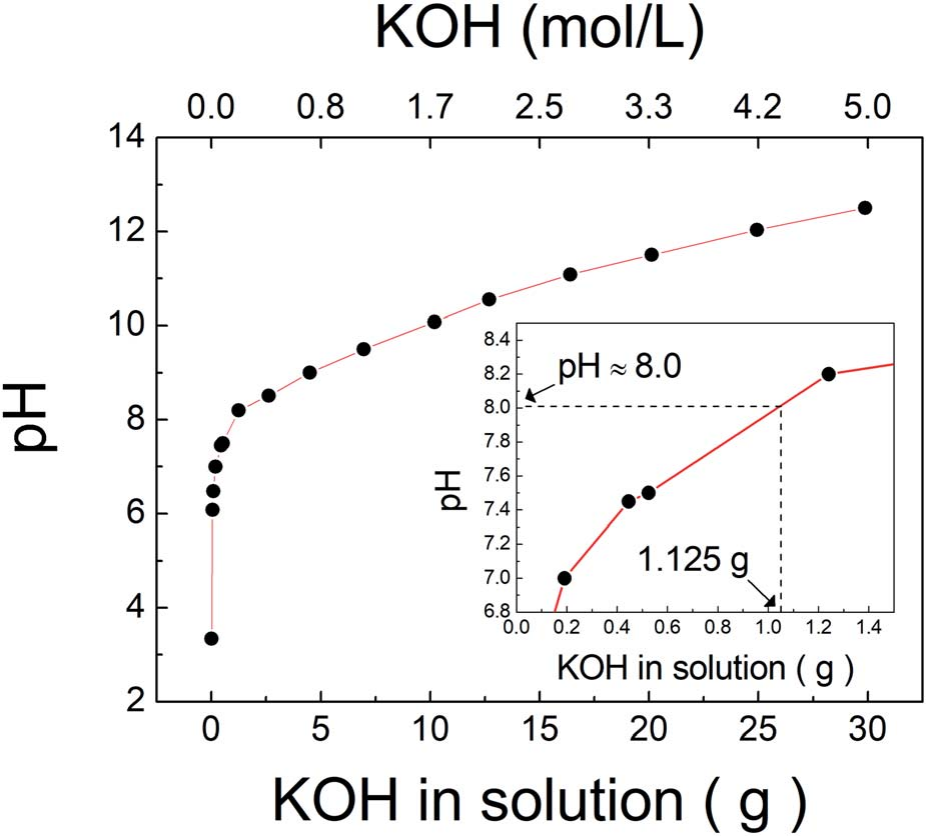
The variation of pH according to the amount and concentration of KOH in solution injected into H_2_O_2_. The inset enlarged range corresponded to 1.125 g of KOH.

The pH is the main factor that affects not only the ionization of TiH_2_, as shown above, but also the number of potassium ions injected for titanate synthesis. Fig. 2 shows the experimental record of the solution pH according to the amount and concentration of KOH when 10 mol L^−1^ of KOH was injected into 62.5 mL of H_2_O_2_. The pH of pure H_2_O_2_ was approximately 3.2, which increased with the injection of KOH. As discussed above, the XRD results indicated that the synthesized compound at pH 8, 10, and 12 was lepidocrocite-type titanate (K_2−*x*_H_*x*_Ti_2_O_5_), which would be represented by a K_2_Ti_2_O_5_ structure assuming that the inter-spaces of titanate for potassium ions are entirely full. Based on this structure, the stoichiometric requirement of KOH for the 0.625 g of TiH_2_ used in the experiment can be calculated to be approximately 1.125 g, which corresponds to approximately pH 8 as shown in the inset of Fig. 2. Because this bottom-up method is based on solution chemical synthesis, adding more potassium ions than theoretically required for the formation of K_2_Ti_2_O_5_ would undoubtedly be beneficial. This may be one factor explaining why the sample at pH 8 had lower crystallinity than the other samples obtained under higher pH conditions. KOH concentrations correspond to 0.02, 0.29, 2.25, and 4.39 mol L^−1^ at pH 6, 8, 10, and 12, respectively. These results show that all of these conditions were obtained at lower KOH concentrations than those required by traditional synthesis methods (≤10 mol L^−1^). The use of an ion precursor is key for the alkali reduction in this chemical bottom-up method.

To determine the effects of the precursor on the synthesis of potassium titanate, we closely examined the traditional precursors; P-25 TiO_2_ and anatase-TiO_2_. First, we applied P-25 TiO_2_ for solution chemical synthesis^29^ under the conditions of 10 mol L^−1^ KOH solution. Although the solution chemical synthesis was carried out at atmospheric pressure, the boiling point of the solution was increased to approximately 113 °C because of the high KOH concentration. The diffraction pattern of the synthesized sample ‘Titanate 1’ is mainly composed of anatase and rutile peak patterns and also shows lepidocrocite-type titanate with very low intensity (Fig. S2, ESI†). The pattern of lepidocrocite-type titanate shows that the intensity increased slightly after the synthetic process, but the peak information is unclear because of the very low and broad shape. Compared to the diffraction pattern of P-25 TiO_2_ used as a precursor, that of rutile shows no noticeable change, but the diffraction intensity of anatase is slightly deceased. Therefore, for the synthesis of layered titanate using KOH, synthetic conditions such as the precursor, temperature, and time need to be modified. Therefore, the hydrothermal method was also performed to synthesize layered titanate using the anatase precursor. The hydrothermal method allows for a synthetic temperature of 160 °C under high-pressure conditions. Fig. S3 (ESI†) shows the XRD pattern of the synthetic results with the hydrothermal method after 3 days (Titanate 2) and 4 days (Titanate 3), which were compared with the pattern of the anatase precursor. Titanate 2 still has the anatase diffraction pattern with high portion under the condition of 3 days. In the case of 4 days (Titanate 3), the anatase pattern reduced significantly, and the pattern of layered titanate is clearly detected. This phenomenon was also confirmed by the SEM images shown in Fig. S4 (ESI†), in which two types of nanostructure were observed. The first type was spherical nanoparticles, as shown in Fig. S4(a) (ESI†); these were attributed to anatase-TiO_2_ particles that remained untreated. The second type was anisotropic nanostructures as shown in Fig. S4(b) (ESI†), which was attributed to layered titanate. The synthesis of layered titanate using KOH depends on the synthetic time, suggesting that not only temperature but also time is a very important factor in the synthesis. Moreover, these results imply that the anatase crystal transformed into layered potassium titanate as the synthesis progressed.

The first influencing factor for the synthesis of potassium titanate may be the type of precursor. The alkali caused fewer changes in the physical characteristics of rutile than those of anatase, which may be because rutile is more stable than anatase.^37^ Therefore, using only anatase powder as a precursor would be beneficial for the synthesis of layered potassium titanate rather than P25-TiO_2_ containing rutile. The second influencing factor may be the synthetic process. In the conventional alkali treatment route, it is known that the Ti–O network of TiO_2_ is broken by hydroxide ions, in which cations are inserted inside the lattice to form a layered alkali titanate structure. The above process of network destruction depends on the alkali strength in the treatment solution. The higher the amount or concentration of alkali hydroxide in the solution or the higher the basicity of the alkali raw material species, the more the destruction reaction of the Ti–O network. On the other hand, when NaOH was used instead of KOH under the same conditions of the alkali concentration (10 mol L^−1^), the lepidocrocite-type titanate structure was clearly synthesized despite synthetic conditions at 115 °C, as shown in Fig. S5 (ESI†). This is well known as Kasuga's method for TNT synthesis.^9^ However, although KOH is a stronger alkali than NaOH, our results show that the synthesis of potassium titanate showed a significantly lower phase conversion yield than the case of sodium titanate, suggesting that it is deeply related to the alkali ion species being intercalated. Oxygen in the broken portion of the Ti–O network is formed in a layered structure while bonding with cations. This bonding process depends on the electrostatic attraction between cations and oxygen in the TiO_*x*_ molecule. Because the strength of electrostatic attraction is inversely related to the inter-nuclear distance between binding atoms, it is also inversely proportional to the size of the ions. When the ionic radius of the cation is bigger, the equilibrium distance between the cation and the anion is longer and the cationic charge density is lower.^38^ The longer distance and the lower charge density give weak electrostatic attraction, resulting in the bonding strength between the TiO_*x*_ molecule and potassium (1.33 Å) possibly being relatively weaker than that of sodium (0.97 Å). This may have to be compensated for by requiring more reaction energy. Therefore, the synthesis of layered potassium titanate requires increasing the temperature and time compared to the synthesis of layered sodium titanate. These results demonstrate that the synthesis of layered titanate can be realized not only at relatively low KOH concentrations but also at low temperature and in a short reaction time through our bottom-up method.

**Fig. 3:**
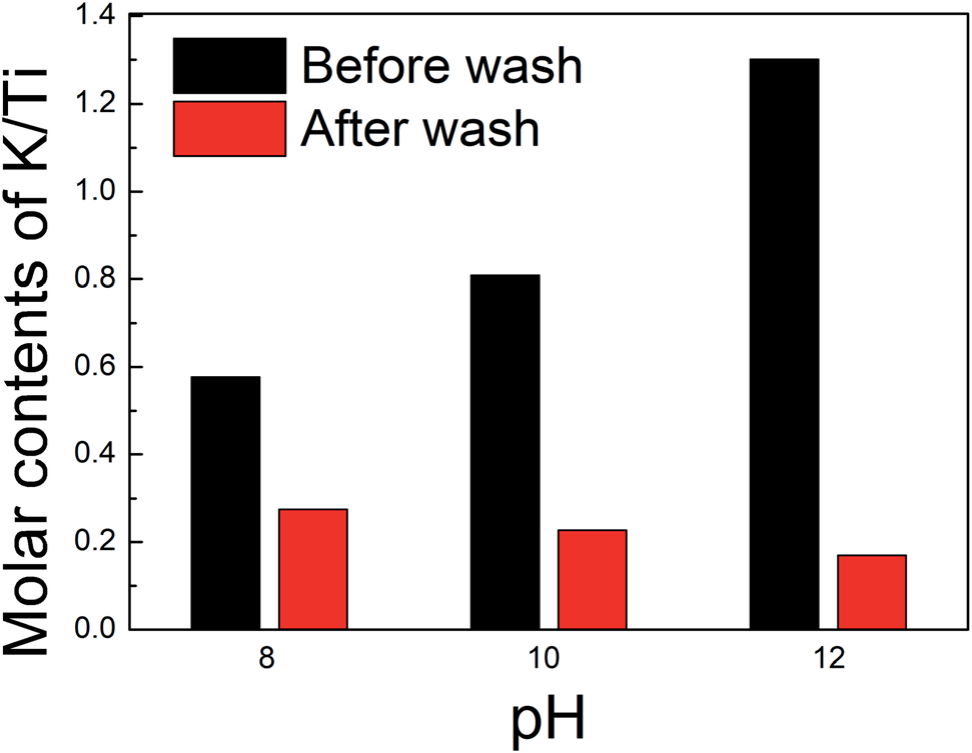
The molar contents of K/Ti in the powder prepared from the peroxo-titanium complex ion at pH 8, 10, and 12, respectively. Black bars display the data of the samples before wash, and red bars display the data of the samples after wash.

The layered titanate structure may depend on the amount of intercalated ions, which can be controlled through the washing process. XRF analysis was performed to predict the amount of potassium ions in the interlayer of titanate. The samples synthesized at pH 8, 10, and 12 were either not washed or washed. Fig. 3 shows the changes in the molar ratios of K/Ti for the samples prepared at each pH depending on the washing process. The molar ratio of K/Ti of unwashed samples increased with the pH, which indicates that an environment rich in potassium ions is favorable for K_2_Ti_2_O_5_ synthesis. However, considering the stoichiometric ratio of K_2_Ti_2_O_5_ (*i.e.*, K/Ti = 1), the results indicated lower molar ratios of 0.56 and 0.80 at pH 8 and 10, respectively, while the molar ratio was as high as 1.32 for pH 12. We obtained similar results in our previous work.^27^ We inferred that the potassium ions were exchanged with protons with a reduced amount, despite the lack of a washing process. The synthetic process took place in a solution containing large amounts of protons, so a spontaneous exchange with potassium ions between layers may occur. The environment of large amounts of potassium ions and hydroxide ions at pH 12 would have disrupted this spontaneous ion exchange. Such a strongly alkaline environment can cause adsorption of the hydroxide molecular (OH^−^) on the titanate surface, which becomes a more negatively charged surface. It would adsorb excess potassium ions other than that contained in the potassium titanate structure, which would have been the cause of the excessive amount of potassium ions as a result of element composition analysis. The washing process further reduced the amount of potassium ions in all samples, which would result in further ion exchange between potassium ions and protons between layers. The resulting K_2−*x*_H_*x*_Ti_2_O_5_ structures at pH 8, 10, and 12 can then be predicted to be K_0.56_H_1.44_Ti_2_O_5_, K_0.44_H_1.56_Ti_2_O_5_, and K_0.32_H_1.68_Ti_2_O_5_, respectively. Notably, the chemical compositions show that the proportion of potassium ions decreased with increasing pH, which would affect the interlayer lattice distance. The ionic radius of a potassium ion is 1.33 Å, which is greater than that of a proton (0.37 Å). Thus, when a potassium ion is exchanged with a proton, the interlayer distance of the structure is reduced. After washing, the potassium content was higher at lower pH (pH 8 > pH 10 > pH 12), and the XRD results indicated that the interlayer distance followed the same trend. Meanwhile, such a layered titanate structure in which protons are inserted is attracting great attention as an electrode material for an efficient and stable proton-based aqueous battery. Recently, Kang *et al.*^39^ demonstrated that this layered compound can exhibit high ionic conductivity both within the interlayer space and titanate layers. Thus, our materials have the function of controlling interlayer properties and are expected to be utilized as promising electrochemical materials by facilitating the movement or storage of ions/molecules.

### Morphological features of titanates synthesized by the bottom-up method

**Fig. 4:**
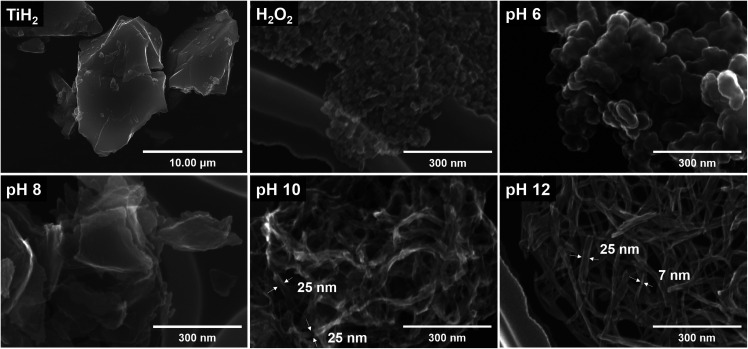
SEM images of commercial TiH_2_ powder and the samples prepared from the ionic precursor at the conditions of H_2_O_2_, pH 6, 8, 10, and 12, respectively.

**Fig. 5:**
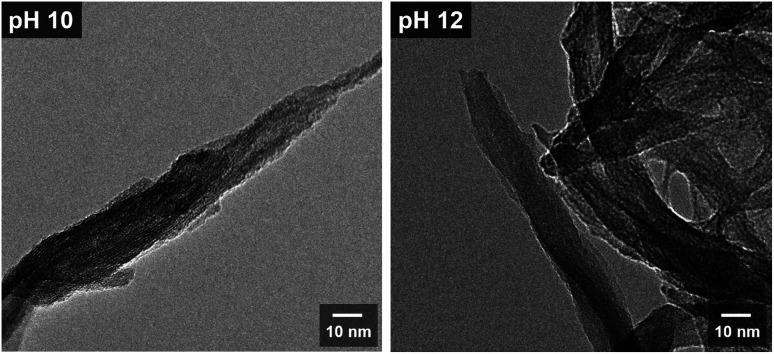
HRTEM images of the samples prepared from the peroxo-titanium complex ion at pH 10 and 12, respectively.

Fig. 4 shows the SEM images of TiH_2_ and the samples synthesized from ionic precursors obtained under various pH conditions. Bulky microstructures were observed for TiH_2_. With H_2_O_2_ and pH 6, nanoparticles agglomerated. The particles were larger at pH 6 (30–70 nm) than those with H_2_O_2_ alone (20–30 nm). Note that the sample labeled as H_2_O_2_ was prepared from a solution containing no KOH. In the case of pH 6, KOH was added but only in a very small amount (0.02 mol L^−1^). At pH 8, stacked sheet-like nanostructures with a width of 200–300 nm were confirmed. This implies that the presence of potassium ions is essential for synthesizing an anisotropic nanostructure derived from layered potassium titanate. At pH 10 and 12, nanostructures with a high aspect ratio were confirmed. Although both samples showed nanoribbon structures with a width of approximately 25 nm and a length of several hundred nanometers, both scrolled and straight structures were confirmed. The magnified HRTEM images in Fig. 5 show that the pH 12 sample had more rigid and straight nanoribbon structures than the pH 10 sample. The pH 10 sample had a tree bark-like pattern on its surface, which may be derived from the washing process based on the chemical composition analysis presented in Fig. 3. Many studies^40–42^ on the formation mechanism of nanotubes have reported that sheet-like structures can be formed by delamination or exfoliation from bulky layered titanate when washed with deionized water, where the alkali ion–proton exchange is a driving force. It was reported that nanosheets can be curved by mechanical tension to decrease excess surface energy during the peeling off process.^42^ Therefore, our nanoribbon structures may have been slightly curved during the process of potassium ion–proton exchange.

**Fig. 6:**
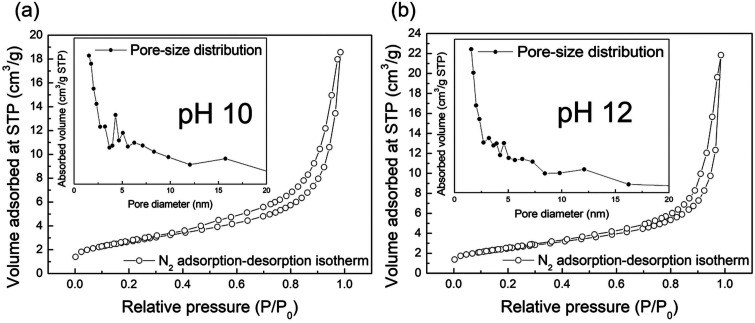
N_2_ adsorption–desorption isotherms of the samples synthesized at pH 10 (a) and 12 (b), respectively. The inset is pore-size distribution of the corresponding samples.

We conducted N_2_ adsorption–desorption isotherm analysis to investigate the specific surface area and pore information of the obtained samples. Fig. 6(a) and (b) show the isotherms at pH 10 and 12, respectively. Both isotherms are type IV with the H3 type hysteresis loop according to the International Union of Pure and Applied Chemistry (IUPAC) classification; this is associated with slit-shaped pores formed by particle agglomeration. The specific surface area and pore volume were 225.829 m^2^ g^−1^ and 0.631 cm^3^ g^−1^, respectively, at pH 10, and 217.426 m^2^ g^−1^ and 0.787 cm^3^ g^−1^, respectively, at pH 12. These results indicate that both samples had similarly high surface areas and pore characteristics originating from the nanoribbon structures, which had a high aspect ratio. We have previously reported the formation of sodium titanate^27^ and lithium titanate^43^ by the bottom-up process using NaOH and LiOH, respectively. These materials show quite different morphological characteristics depending on the type of alkali species used. In the case of using LiOH, the nanoplate structure with a low aspect ratio was formed, while the nanotube structure was formed in the case of using NaOH, which showed a lower specific surface area as compared with KOH. The contribution of alkali species to their aspect ratio was in the order of KOH > NaOH > LiOH, which basically accorded with the order of the ion radius K^+^ > Na^+^ > Li^+^. Although the Na^+^, K^+^, and Li^+^ ions have the same positive charge as the same monovalent cation, it is expected that oxygen atoms of TiO_*x*_ molecules would be less attracted to K^+^ as compared with Na^+^ or Li^+^ during the crystal growth as already mentioned above. This reduces the bonding strength between layers and promotes anisotropic crystal growth along the same layer direction rather than the interlayer direction, forming a nanostructure with a high aspect ratio and large specific surface area in the case of using KOH. Typically, it is known that the photocatalytic activity is related to the surface area and improves as the surface area of the structure increases. A larger specific surface area can involve more active sites and adsorption area for the substance.^44^ The insets of each figure show the pore size distribution. At pH 10, the noticeable characteristic pore size was found at approximately 4.3 nm, contrary to the case of pH 12, which might have originated from the pores formed from scrolled nanostructures.

### Optical properties of titanates

**Fig. 7:**
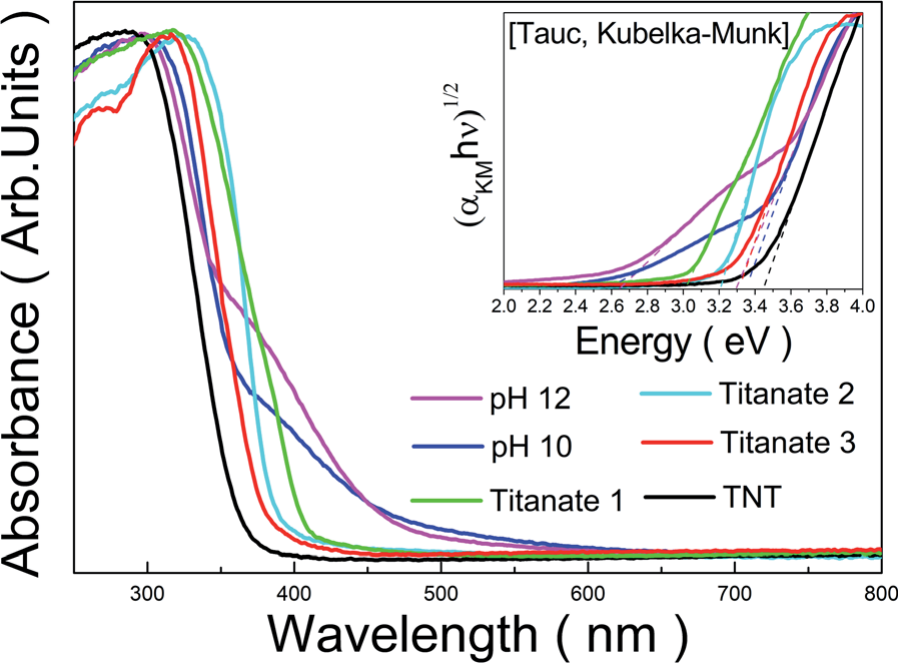
Absorbance spectra of titanate prepared by various methods. The inset shows Tauc plots to observe the optical band-gap energy corresponding to the samples.

The optical properties of the prepared samples were investigated by UV-Vis analysis and were compared with those of TNTs composed of a traditional hydrogen titanate structure, as shown in Fig. 7. The samples from bottom-up synthesis with the peroxo-titanium complex ion precursor at pH 10 and 12 were labeled as “pH 10” and “pH 12”, respectively, to avoid confusion with the other titanates. The absorbance spectrum of the TNTs showed an absorption edge at around a wavelength of 360 nm. For Titanate 1 (solution chemical synthesis with KOH), the absorption edge was observed at around 410 nm, which is a slightly longer wavelength than that for TNT. For Titanate 2 and Titanate 3 (hydrothermal method), increasing the titanate phase in the structure shifted the absorbance characteristics to lower wavelengths (*i.e.*, blue shift). The absorption edges for Titanate 2 and 3 were at around 400 and 380 nm, respectively. For the pH 10 and pH 12 samples, the absorbance edges were at around 470 and 480 nm, respectively. Thus, better absorbance characteristics were obtained with the peroxo-titanium complex ion precursor than with the other methods.

DRS analysis was used to measure the reflectance of the titanates, which was converted into the bandgap energy by the Tauc Kubelka–Munk method. The results are presented in Table 1. The TNTs had a bandgap of 3.45 eV, which is similar to that reported for hydrogen titanate elsewhere. Titanate 1 had a bandgap of 3.04 eV; the difference was attributed to the presence of the rutile phase, which has a bandgap of approximately 3.00 eV.^45^ For Titanate 2 and 3, the bandgaps widened to 3.22 and 3.32 eV, respectively, which indicates that the bandgap increased with the titanate phase. In contrast, the pH 10 and pH 12 samples showed quite narrow bandgaps of 2.59 and 2.69 eV, respectively. Both bandgaps are very similar to that reported previously for peroxo-titanate nanotubes (2.50 eV).^4^ We attributed the visible light activation to the peroxo-titanium bonds (Ti–O–O). Although the bandgap for pH 10 was lower than that of pH 12, the case for pH 12 had better absorbance over a wider area of the spectrum. However, for both samples in the present study, another optical absorption edge can be seen in the absorption curve at around 375 nm. These can also be found as another linear region in the Tauc plot, and correspond to the band gaps of 3.35 and 3.29 eV in the case of pH 10 and pH 12 samples, respectively. These values are considered to be derived from the band gap of intrinsic titanate materials that do not contain peroxo-titanium bonds.

**Table 1:** Band-gap energy of titanates prepared by various methods.

Sample	pH 12	pH 10	Titanate 1	Titanate 2	Titanate 3	TNT
Band-gap (eV)	2.69	2.59	3.04	3.22	3.32	3.45

**Fig. 8:**
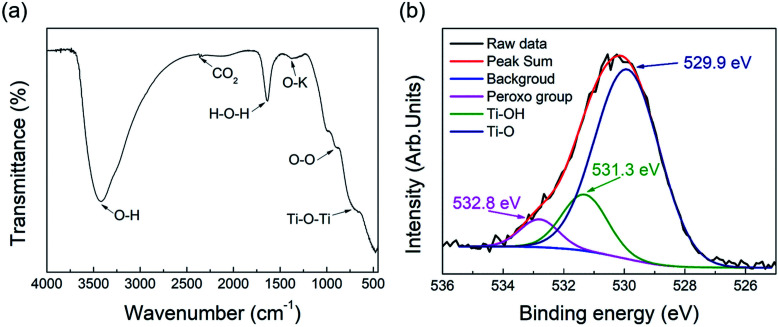
(a) FT-IR and (b) O_1s_ XPS spectra of the pH 12 sample.

The bond characteristics of the pH 12 sample, which showed the best response to visible light among the samples, were investigated by FT-IR and XPS analyses. The FT-IR spectrum in Fig. 8(a) shows peaks at 3400, 2358, and 1640 cm^−1^, which were attributed to the O–H stretching vibration, CO_2_ vibration, and H–O–H binding vibration, respectively.^46,47^ The broad peak at 700 cm^−1^ was attributed to the Ti–O–Ti vibration.^48^ Cations are connected to oxygen atoms of the TiO_6_ octahedron between layers of the layered titanate crystal, which was reflected in the FT-IR spectrum.^49^ The layered titanate with interlayer sodium ions showed a peak at 1353 cm^−1^, which was attributed to the O–Na stretching vibration.^49^ Therefore, the peak at 1380 cm^−1^ in the pH 12 sample may be due to the O–K stretching vibration. Fig. S6 (ESI†) shows the FT-IR spectrum of the pH 12 sample before washing. A stronger peak was observed at the same position of 1380 cm^−1^, which indicates that the peak intensity could decrease as potassium ions are removed by washing. FT-IR analysis has been used to detect the peroxo bond (O–O) in many studies.^50–53^ Our results also indicated the presence of the O–O bond at 900 cm^−1^, as shown in Fig. 8(a). However, the presence of the peroxo group may be limited by the detection resolution of FT-IR because of its weak detection signal, which can also be confirmed by further XPS analysis. Fig. 8(b) shows the O_1s_ XPS spectra, where the pattern was separated into three peaks assigned to the Ti–O, Ti–OH, and peroxo groups (O–O) at 529.9, 531.3, and 532.8 eV, respectively.^54^ In contrast, the O_1s_ XPS peaks of typical titanate materials have been reported to be separated by only two peaks, and the absence of the peak corresponds to the peroxo groups.^4,54^ The proportion contribution of the separated peak assigned to the peroxo group is 5.41% in the case of pH 12 sample. Until now, several ways to reduce the bandgap energy of titanate have been reported in many studies.^55–57^ These methods have been mainly based on the control of the titanium ion environment, in which the process of reducing Ti^4+^ to Ti^3+^ creates an intermediate-energy defect state between the valence band and conduction band.^58^ The created gap enhances the absorption of visible light and excitation of photogenerated electrons. Fig. S7 (ESI†) shows the Ti_2p_ XPS spectra of the pH 12 sample, where only two peaks were assigned to Ti_2p3/2_ and Ti_2p1/2_ at 457.8 and 463.5 eV with 5.7 eV splitting.^59^ Our results demonstrate the bandgap reducing method through the ligand control of titanium molecules (*i.e.*, Ti–O–O bond formation), which is different from previous methods.

### Photocatalytic properties of titanates

**Fig. 9:**
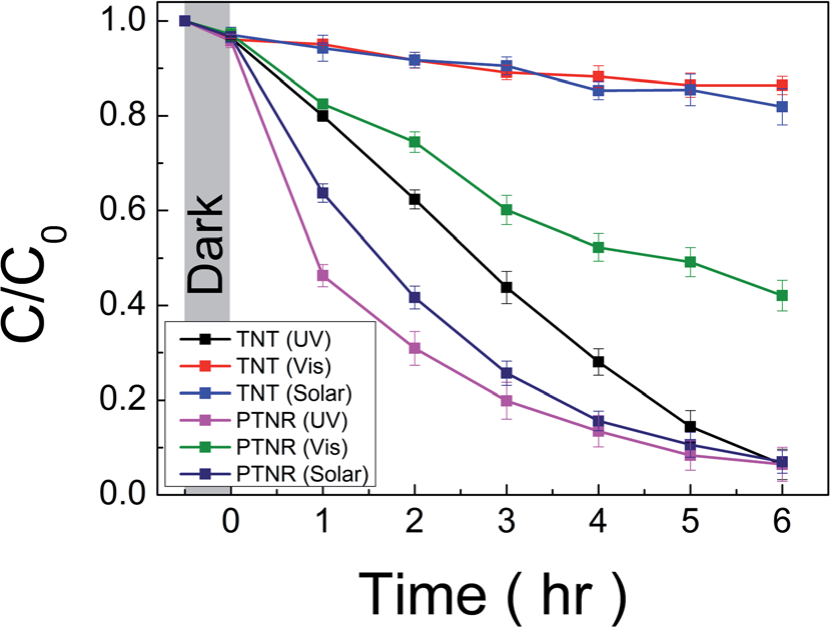
Normalized intensity (*C*/*C*_0_) of RhB dye with respect to incident light irradiation time in the presence of TNT and PTNR under UV, visible, and solar lights.

The photocatalytic performance was investigated through RhB decolorization under UV (<380 nm), visible light (>420 nm), and solar irradiations. The wavelength of UV and visible lights was controlled as described in the Experimental section and as shown in Fig. S1 (ESI†). To confirm the functionalization of the photocatalytic performance in each wavelength region, the pH 12 sample, which we called a peroxo-titanate nanoribbon (PTNR), was compared with TNT. TNT was selected as a reference sample based on the similar morphology, specific surface area (239.519 m^2^ g^−1^), and crystal phase to pristine layered titanate, as shown in Fig. S5 and S8 (ESI†). Fig. 9 shows the photocatalytic performance (*C*/*C*_0_) *versus* the irradiation time for the RhB decolorization. Under UV light irradiation, both the TNT (UV) and PTNR (UV) showed excellent photocatalytic reactions of 93.5%, compared to the initial concentration over 6 h of reaction time. However, both catalytic kinetics of decolorization reactions are found to be slightly different. Hence, the Langmuir–Hinshelwood (L–H) first-order reaction dynamic model was introduced to compare photocatalytic efficiency more accurately as shown in Fig. S9 (ESI), and Table S3 (ESI†) summarizes the decolorization, calculated rate constant (*K*_app_), and *R*^2^ values for each condition. The calculated *K*_app_ value of TNT (UV) is 5.53 × 10^−3^ min^−1^ and that of PTNR (UV) is 6.91 × 10^−3^ min^−1^. The kinetic value might be increased due to the high RhB adsorption properties. Although the TNT had a similar specific surface area to the PTNR, the more negative potential of the peroxo-bond can make the adsorption of cationic RhB dye easier compared with the intrinsic Ti–O bond on the surface.^4^ Under visible light irradiation, the TNT (Vis) showed a photocatalytic reaction of 13.6% over 6 h of reaction time. The TNT had difficulty with absorbing and utilizing light above wavelengths of 420 nm as discussed in the Introduction section. On the other hand, the PTNR, which can utilize visible light because of the presence of peroxo-titanium bonds, showed a better photocatalytic performance of 57.9% at the sample of PTNR (Vis). Over 6 h of photocatalytic reaction, the PTNR showed a reactivity approximately 1.61 times better under UV light than under visible light. The energy of the light utilized by the PTNR can be calculated from the integral of the overlapping areas of the used light spectra (Fig. S1, ESI†) and its absorption spectrum (Fig. 7). The calculation results showed that the PTNR had a theoretical absorption energy of 2220 W m^−2^ for UV light and 1500 W m^−2^ for visible light. Interestingly, this indicates that the PTNR does approximately 1.48 times better with UV light than with visible light, which is very similar to the relationship for the photocatalytic reactivity. Similar to this, we have already reported that photocatalytic properties under visible light can be enhanced by band gap reduction originating from the peroxo-titanium bond in the case of sodium titanate (PTNT; peroxo-titanate nanotube).^4^ Although the PTNR has a higher specific surface area of 217.4 m^2^ g^−1^, better performance under visible light illumination was not achieved compared with PTNT (183.1 m^2^ g^−1^). We assume that light absorption is one of the most dominant factors in the photocatalytic reaction under visible light. The PTNR displays a slightly lower light absorption in the visible light range and a wider band gap energy compared with the PTNT as shown in Fig. S10 (ESI†). The visible light activation revealed in the peroxo-titanate compounds has been considered to be due to the presence of peroxo-bonds, thus the light absorption rate in the visible light may depend on the amount of peroxo-bonds present in the structure. In fact, the proportion contributions of the peroxo bond at the peaks associated with the oxygen species analyzed by XPS were 5.41 and 11.54% in the cases of the PTNR and PTNT,^4^ respectively. Although the factors that determine the amount of peroxo-bonds to be modified in the titanate crystal could not be fully understood, the present work proved the function of band energy control through modifying a functional ligand in titanate compounds. Their photocatalytic performance under the solar light was also evaluated. The irradiation spectrum of solar light contained a higher wavelength range of 350–400 nm compared with the case of visible light irradiation using a band-pass filter as shown in Fig. S1 (ESI†). For over 6 h of photocatalytic reaction, the TNT (solar) shows a decolorization reaction of 18.1%. Considering the characteristics of light absorption, since TNT has lower light absorption properties in the range of 350–400 nm, only a 4.5% increase was recorded. On the other hand, the PTNR shows a decolorization reaction of 93.0%. The PTNR has better light absorption properties in the corresponding wavelength range, enhancing photocatalytic performance compared to that when the visible light was irradiated.

The stability or recyclability of the photocatalyst is an important factor in their practical utilization. To test the suitability of photocatalytic performance of the PTNR sample under visible light irradiation, the recycling photocatalytic test was carried out as shown in Fig. S11 (ESI†). After 3 cycles, the reusability of the PTNR has no significant decrease in photocatalytic performance, indicating that the PTNR can be successfully reused and stable under visible light. These results demonstrate that extending the light absorption region can enhance photocatalytic performance and result in a highly efficient and stable photocatalyst material.

**Fig. 10:**
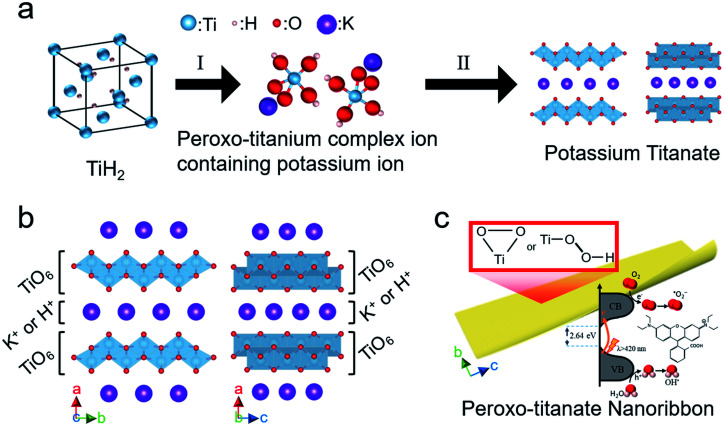
(a) A schematic of the proposed bottom-up synthesis route for peroxo-titanate nanoribbons; (I) formation of the peroxo-titanium complex ion containing potassium ions from TiH_2_ powder by treating with H_2_O_2_/KOH solution. (II) Formation of layered titanate from peroxo-titanium complex ions. (b) Hypothetical schematic crystal structures along [001] and [010] of hydrogenated potassium titanate. (c) Formation of peroxo-titanate nanoribbons with enhanced photocatalytic activity.

Fig. 10 shows the flow of the proposed bottom-up synthesis of visible light-activated PTNRs. In step I (Fig. 10(a)), TiH_2_ is dissolved in an aqueous solution of H_2_O_2_ and KOH and is then converted into a peroxo-titanium complex ion containing potassium ions. This step is accelerated by increasing pH, which increases the supply of potassium ions. The peroxo-titanium complex ion containing potassium ions was clearly formed at pH 8–12 (0.29–4.39 mol L^−1^ KOH). In step II, the prepared complex ion was crystallized by self-organization into potassium titanate by solution chemical synthesis at 100 °C for 24 h without additional alkali input, which was achieved at a relatively low temperature, low pressure, and in a short time compared with previous reports.^19–21^ This can be attributed to two reasons: no destruction of Ti–O bonds, and no insertion of potassium ions into the TiO_2_ crystal lattice. The ionic precursor can combine with potassium titanate crystals through self-organization of the titanium and potassium ions. A crystallographic structure of K_2−*x*_H_*x*_Ti_2_O_5_ (*i.e.*, hydrogenated potassium dititanate) results in layered titanate in which TiO_6_ octahedra are combined by sharing edges,^60^ as shown in Fig. 10(b). The lattice is orthorhombic, and the TiO_6_ layers are laminated with an alternating interlayer of cations (K^+^ or H^+^) along the *a*-axis direction.^61^ Bavykin *et al.*^22^ reported that titanate nanowires or nanofibers grow along the *c*-axis direction. At pH 10 and 12, where the supply of potassium ions is sufficient, PTNRs with a high aspect ratio were formed with further crystal growth, as shown in Fig. 10(c). Note that the peroxo-titanium bond of the complex ion formed in step I of Fig. 10(a) transfers to the final layered titanate structure through step II. The presence of the peroxo-titanium bond decreases the surrounding electron density of the titanium atoms, which narrows the bandgap of the titanate.^51^ The reduced bandgap allows the electron to be easily excited by photons even under visible light. The resultant electron hole pairs (e^−^/h^+^) may migrate to form active radicals such as the superoxide radical (˙O_2_^−^) and hydroxyl radical (OH˙) that exhibit strong oxidation of organic matters such as RhB dye. Therefore, detailed analysis of active species under the various irradiations is required to evaluate PTNR as a photocatalyst. The present study demonstrated that the crystal structure and morphology of the product can be controlled simply by replacing the alkali species in the chemical synthesis route. Accordingly, through the bottom-up process using K^+^, it was possible to synthesize titanate nanoribbon structures with a large specific surface area and excellent light response.

## Conclusions

The peroxo-titanium complex ion was successfully applied as a precursor for the bottom-up synthesis of PTNRs under low-alkali and low-temperature conditions. The ionic precursor was prepared at various pH values to determine the optimal conditions for the ionization of TiH_2_ and synthesis of layered titanate structures. The results showed that increasing pH promoted the ionization of TiH_2_ to form the peroxo-titanium complex ion and provided the potassium ions required for layered titanate structures. The formation of layered titanate strongly depended on the constituent materials and synthetic conditions. Our experiments demonstrated that the amount of KOH and synthetic temperature can be drastically lowered when the peroxo-titanium complex ions are used as precursors by replacing the solid-crystal titania. Moreover, the use of potassium-contained peroxo-titanium complex ion precursor was advantageous for the anisotropic crystal growth for the synthesis of nanostructures with a high aspect ratio than sodium or lithium-based ion precursors. The optical and structural analyses revealed that the PTNR contains a peroxo-titanium group that enhances the optical response to the visible light. The RhB decolorization test demonstrated that the PTNR has excellent photocatalytic properties under UV, Vis, and solar light irradiations based on its high specific surface area and excellent visible light activity. In this work, we presented a simple and effective method for synthesizing titanate nanostructures based on self-organization, which may also be applicable to the synthesis of other structures with advanced chemical and photochemical properties.

## Author contributions

HP: conceptualization, methodology, investigation, formal analysis, investigation, data curation, and writing – original draft. DHH, TG, SC, and YM: methodology, investigation, data curation, and writing – review & editing. TS: supervision, conceptualization, methodology, formal analysis, investigation, data curation, resources, funding acquisition, and writing – review & editing. All authors discussed the results and the implications of this manuscript. All authors have given approval to the final version of the manuscript.

## Conflicts of interest

The authors declare no competing financial interest.

## Supplementary Material

NA-004-D2NA00234E-s001
