# Supplementary material for: A facile bottom-up method for synthesis of peroxo-potassium titanate nanoribbons and visible light photocatalytic activity derived from a peroxo-titanium bond
Source: Nanoscale Adv. 2022 Jul 20;4(17):3573–84. doi: 10.1039/d2na00234e (PMC9400506; doi:10.1039/d2na00234e)
Supplement: NA-004-D2NA00234E-s001 [file NA-004-D2NA00234E-s001.pdf]

Electronic Supplementary Material (ESI) for Nanoscale Advances.
This journal is © The Royal Society of Chemistry 2022

Supplementary information for:

Facile bottom-up method for synthesis of peroxo-potassium titanate nanoribbons and visible light photocatalytic activity derived by peroxo-titanium bond

Hyunsu Park,[a] Do Hyung Han,[a] Tomoyo Goto,[a,b] Sunghun Cho,[a] Yukihiro Morimoto,[a] and Tohru Sekino*[a]*

[a]SANKEN (The Institute of Scientific and Industrial Research), Osaka University, 8-1 Mihogaoka, Ibaraki, Osaka, 567-0047, Japan

[b]Institute for Advanced Co-Creation Studies, Osaka University, 1-1 Yamadaoka, Suita, Osaka, 565-0871, Japan.

*E-mail: hspark23@sanken.osaka-u.ac.jp, sekino@sanken.osaka-u.ac.jp

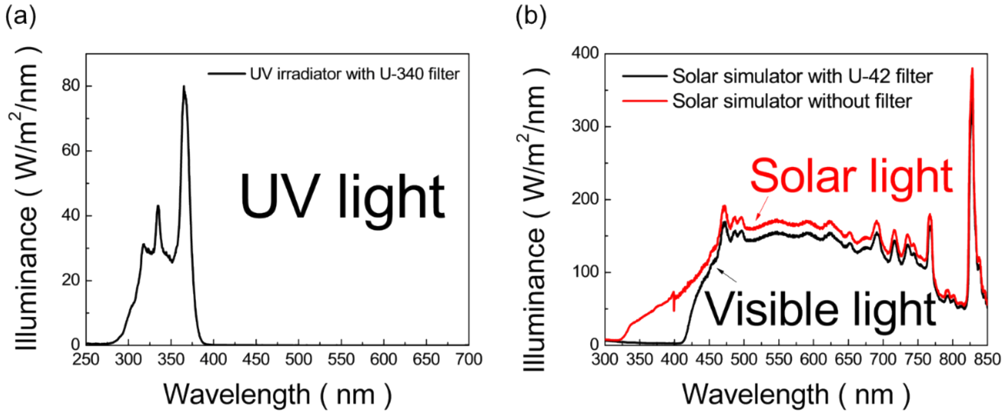

Fig. S1 The kind of illuminance spectra used in photocatalytic test: (a) UV light prepared by UV irradiator with U340 bandpass filter, which illuminates light between 280 and 380 nm of wavelength. (b) visible light was obtained by eliminating the light less than 420 nm of wavelength using a cut filter L-42 from the solar light irradiated form the solar simulator. In the case of solar light, the filter was not used.

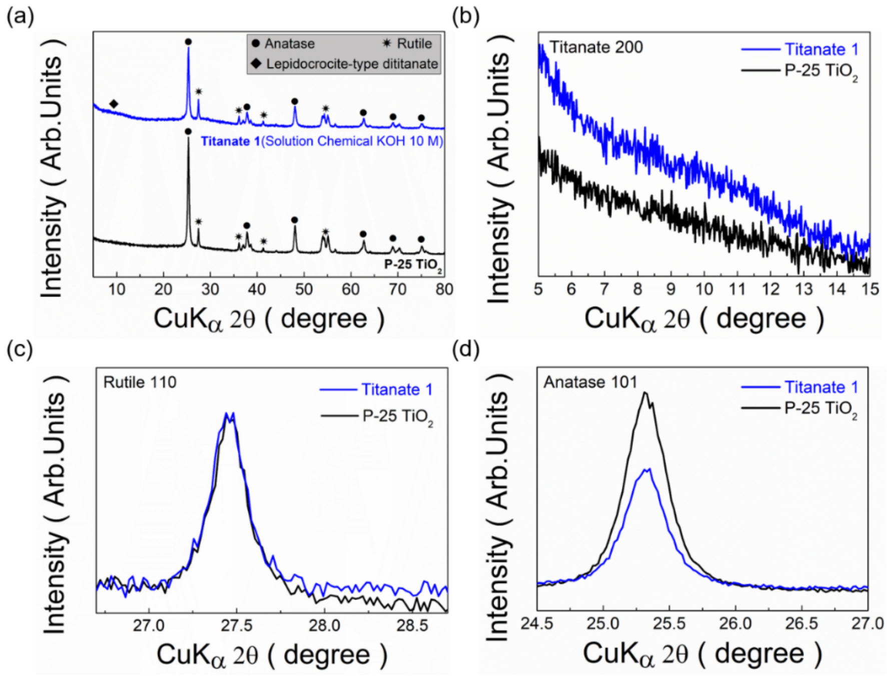

Fig. S2 (a) XRD patterns of arising from powders synthesized by solution chemical method under 10 mol/L of KOH solution at 113 °C for 24 h using P25-TiO_2 precursor, and commercial P25-TiO_2 powder. Enlarged diffraction ranges around where (b) 200 peak of lepidocrocite-type titanate, (c) 110 peak of rutile, and (d) 101 peak of anatase, respectively. The intensity of the 101 peak of anatase decreased by approximately 35.5 % as shown in (d).

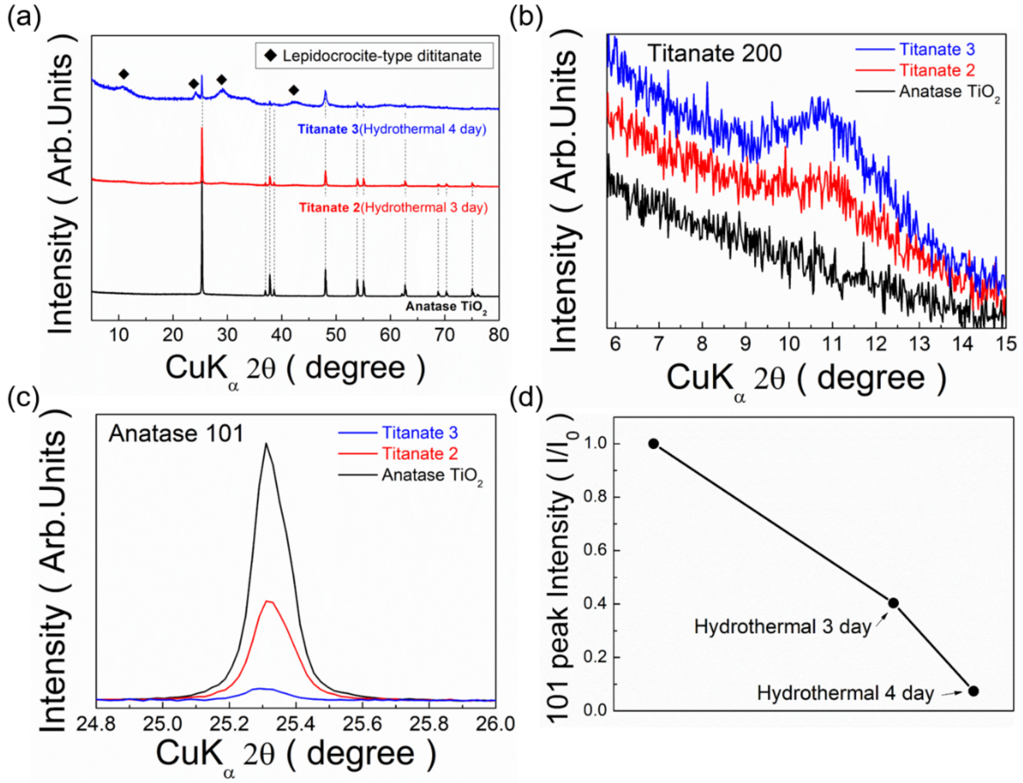

Fig. S3 (a) XRD patterns of arising from powders synthesized by hydrothermal method under 10 mol/L of KOH solution at 160 °C for 3 and 4days using anatase-TiO_2 precursor, and commercial anatase-TiO_2 powder. Enlarged diffraction ranges around where (b) 200 peak of potassium titanate and (c) 101 peak of anatase. (d) Variation of relative peak intensity on anatase 101 peak using the formula: I/I_0, where, I_0 and I were peak intensity corresponding 101 peak of anatase in the case of anatase precursor and synthesized sample, respectively, it is plotted according to synthetic time. The intensity decreased linearly by approximately 59.6% and 92.6% according to the 3 and 4 days synthetic times, respectively.

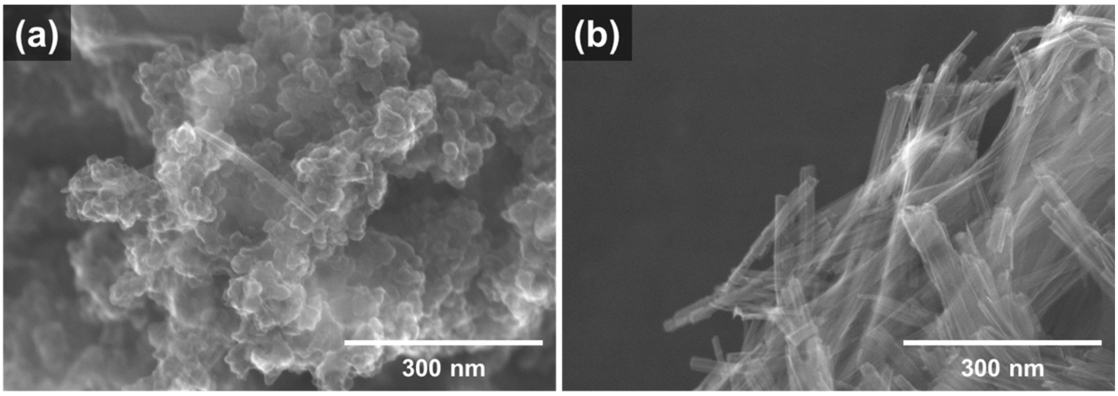

Fig. S4 SEM images of morphology types observed in particles treated under 10 mol/L of KOH at 160 °C for 4 days as discussed in Fig. S3. (a) image shows the relatively spherical shape of nanoparticles and (b) image shows the relatively anisotropic nanostructures.

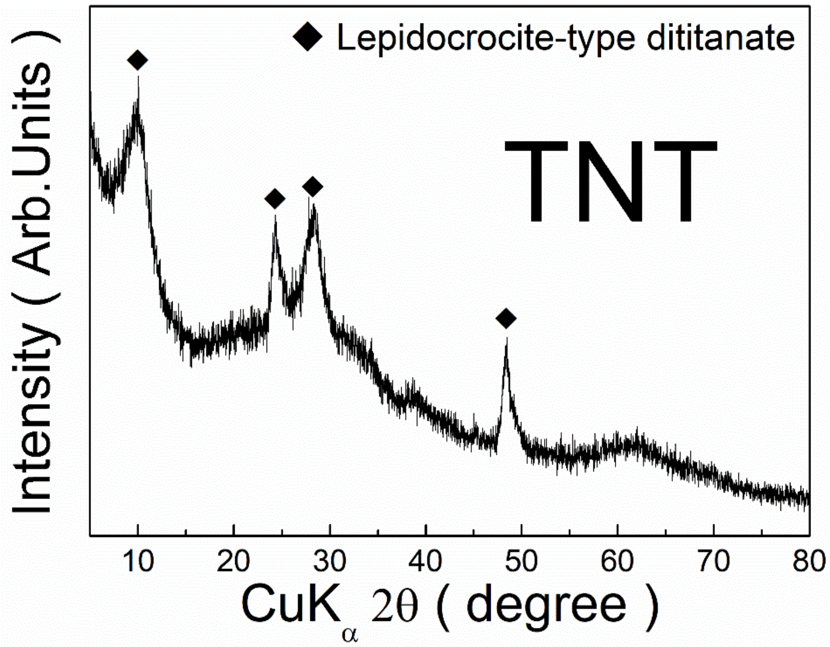

Fig. S5 XRD pattern of TNT synthesized by Kasuga's method under 10 mol/L of NaOH solution at 115 °C for 24 h. The pattern mainly assigned to lepidocrocite-type dititanate.

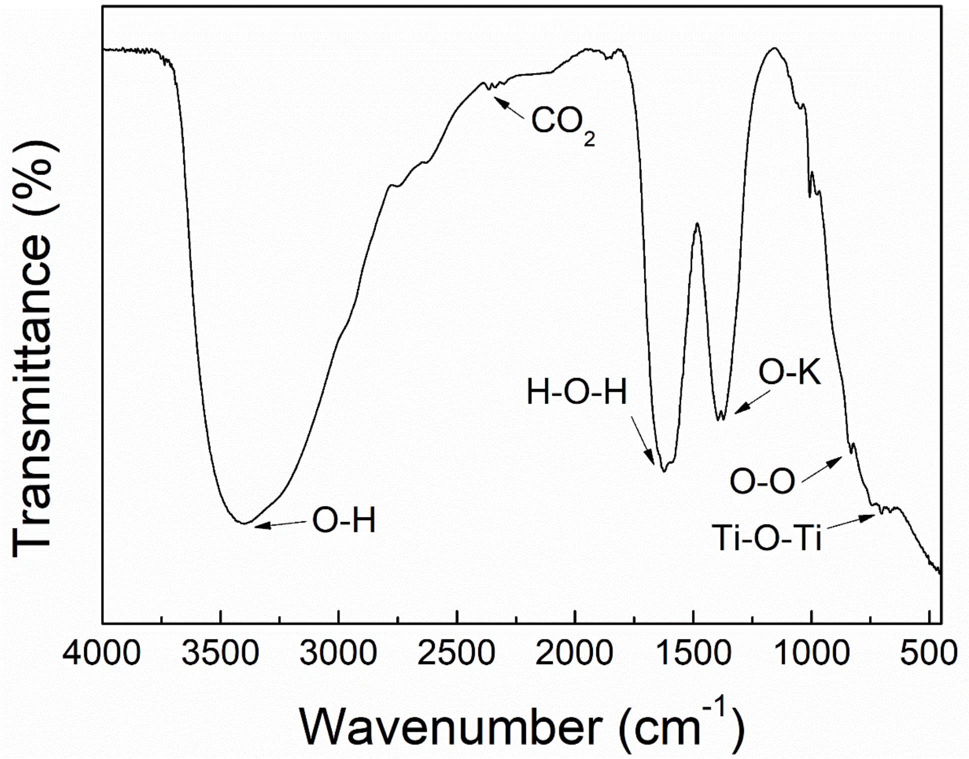

Fig. S6 FT-IR spectrum of pH 12 sample obtained before washing.

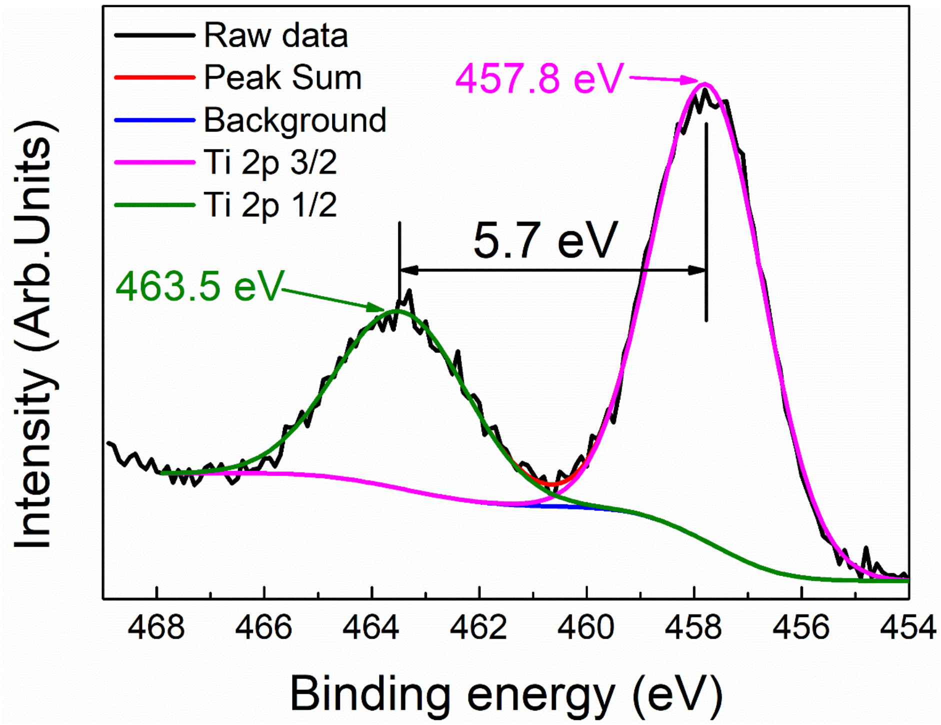

Fig. S7 XPS spectra in the range of pH 12 sample.

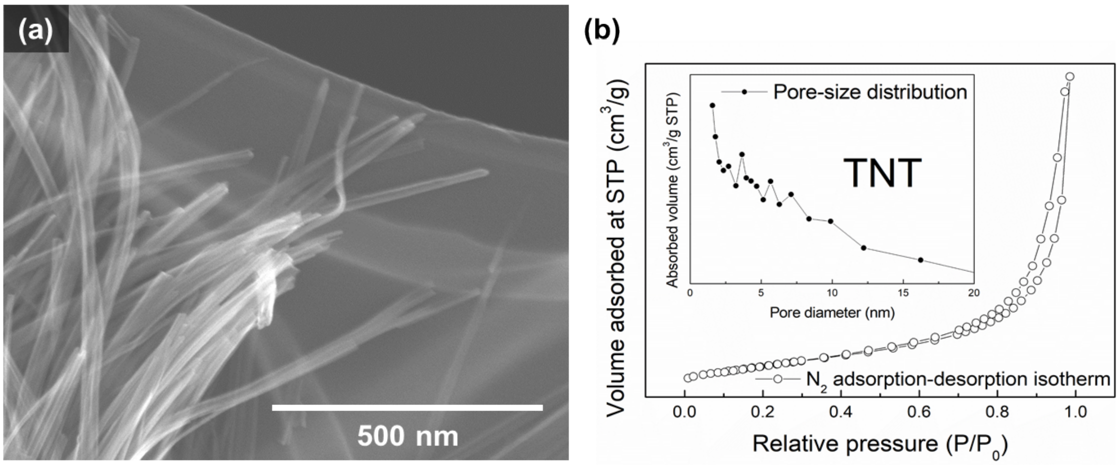

Fig. S8 (a) SEM image and (b) N_2 adsorption-desorption isotherm containing inset of pore-size distribution graph of TNT. The isotherm is type IV with the H3 type hysteresis loop according to the International Union of Pure and Applied Chemistry (IUPAC) classification; this is associated with slit-shaped pores formed by particle agglomeration. The specific surface area and pore volume are 239.519 m^2/g and 1.005 cm^3/g, respectively.

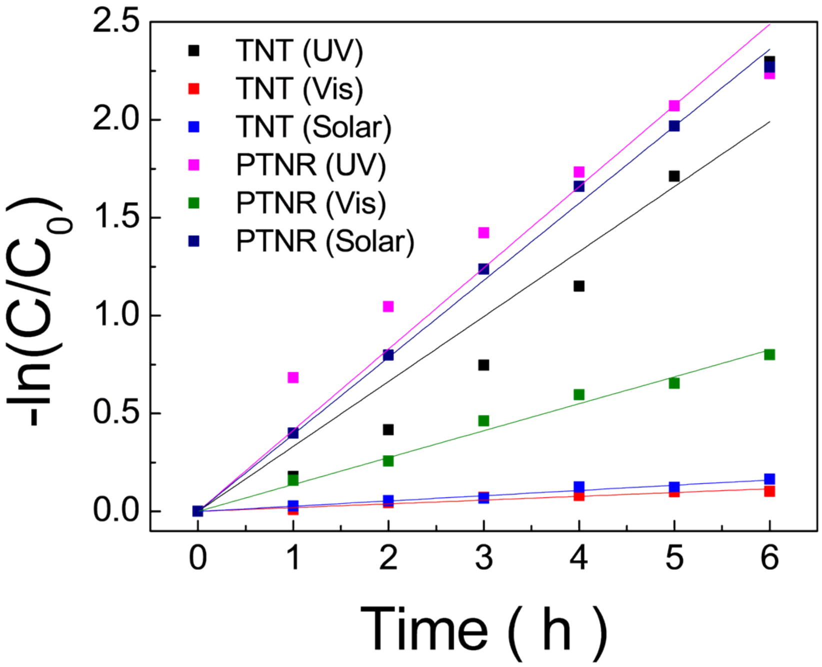

Fig. S9 First-order linear transformation $-\ln(C/C_0)$ plot with respect to incident light irradiation time in the presence of TNT and PTNR under UV, Visible, and solar lights.

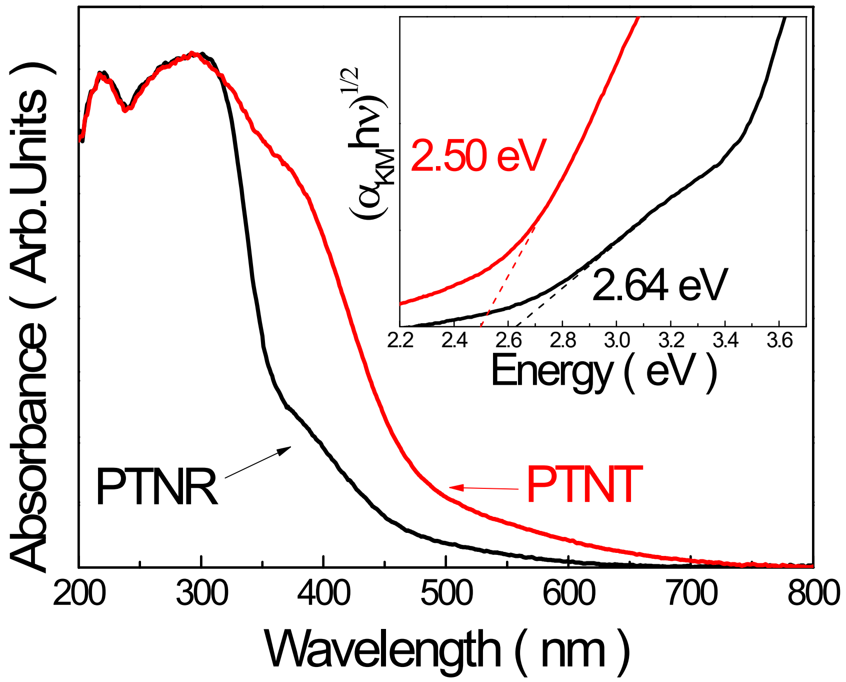

Fig. S10 Absorbance spectra of PTNR and PTNT. The inset shows Tauc plots to observe the optical band-gap energy corresponding to the samples

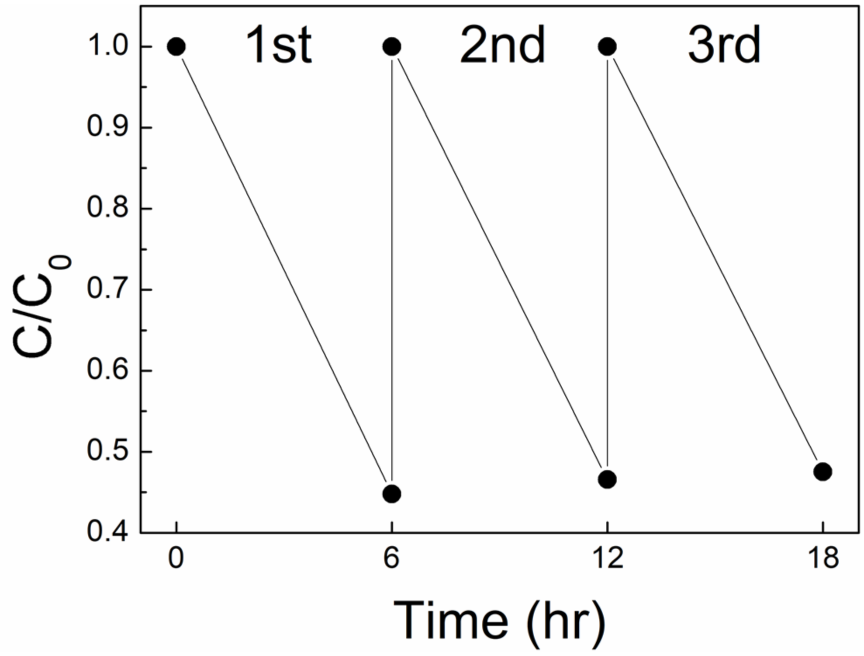

Fig. S11 Recycling photocatalytic test of PTNR under visible light. After each cycle, the RhB solution was added for the concentration correction. The decolorization reactions of RhB (C/C_0) were recorded to 0.45, 0.47, and 0.48 after 1, 2, and 3 cycles, respectively.

Table S1 Comparison of previous works for formation of potassium titanate

Year	Morphology (size)	Precursor	Synthetic conditions	Reference
2002	Nanobelt	TiO_2, KOH(4–10M)	Liquid 160–180 °C, 48 h	(1)
2003	Nanowire(10 nm, 500 nm–2 μm)	TiO_2, KOH(10M)	Liquid, 130 °C, 3 days	(2)
2007	Partially Nanotube	TiO_2, KOH(10M)	Liquid, 56 °C, 12 days	(3)
2011	Nanoribbon	TiO_2 (P-25), KOH(5–18M)	Liquid, 120–150 °C, 24 h	(4)
2011	Nanoribbon (5–8 nm)	TiO_2 (P-25), KOH(10M)	Liquid, 200 °C, 1 h	(5)
2012	Nanoribbon (15–50 nm, >800 nm)	TiO_2 (Anatase), KOH(10M)	Liquid, 170–200 °C, 96 h	(6)
2012	Nanorod (5–10 nm, 100 nm)	Titanium hydroxide, KOH(10 M)	Liquid, 180 °C, 24 h	(7)
2015	Nanotube	TiO_2, NaOH, KOH(9:1, 10M)	Liquid, 100–160 °C, 20 h	(8)
2017	Nanoribbon (20nm, several μm)	TiO_2 (Anatase), KOH(10M)	Liquid, 160 °C, 4 day	(9)
2018	Nanoribbon (200 nm, >30 μm)	TiO_2, K_2CO_3	Solid 1250 °C	(10)
2019	Nanowire (10 nm, Several hundreds of nm)	TiO_2, K_2CO_3, Li_2CO_3 (2.4:0.8:10.4)	Solid 600 °C, 20 h	(11)

Table S2 Sample list in this study

Notation	Materials	Alkali concentration	Synthetic conditions
pH 6	TiH_2, H_2O_2, KOH	0.02 M	100 °C, 24 h
pH 8	TiH_2, H_2O_2, KOH	0.29 M	100 °C, 24 h
pH 10	TiH_2, H_2O_2, KOH	2.25 M	100 °C, 24 h
pH 12	TiH_2, H_2O_2, KOH	4.39 M	100 °C, 24 h
Titanate 1	TiO_2 (P-25), KOH	10 M	113 °C, 24 h
Titanate 2	TiO_2 (Anatase), KOH	10 M	Hydrothermal

			160 °C, 3 days
Titanate 3	TiO_2 (Anatase), KOH	10 M	Hydrothermal<br>160 °C, 4 days
TNT	TiO_2 (P-25), NaOH	10 M	115 °C, 24 h

Table S3 RhB decolorization, Kapp value calculated from the pseudo-first-order Langmuir-Hinshelwood equation.

	TNT (UV)	TNT (Vis)	TNT (Solar)	PTNR (UV)	PTNR (Vis)	PTNR (solar)
Decolorization (%)	93.5	13.6	18.1	93.5	57.9	93.0
K_{app} (min^{-1})	0.005529	0.000322	0.000446	0.006912	0.002291	0.006556
R^2	0.9356	0.9448	0.9709	0.9424	0.9857	0.9954

REFERENCES

1 X. Sun, X. Chen and Y. Li, *Inorg. Chem.*, 2002, **41**, 4996–4998.

2 Y. Yu, P. D. Han, L. M. Peng, G. H. Du and Q. Chen, *Phys. Rev. B - Condens. Matter Mater. Phys.*, 2003, **67**, 035323.

3 D. V. Bavykin, B. A. Cressey and F. C. Walsh, *Aust. J. Chem.*, 2007, **60**, 95.

4 L. M. Sikhwivhilu, S. Sinha Ray and N. J. Coville, *Appl. Phys. A Mater. Sci. Process.*, 2009, **94**, 963–973.

5 Q. Li, T. Kako and J. Ye, *Int. J. Hydrogen Energy*, 2011, **36**, 4716–4723.

6 J. Xu, H. Zhang, W. Li, J. Zhang, X. Liu, X. He, D. Xu, J. Qian and L. Liu, *Micro Nano Lett.*, 2012, **7**, 654–657.

7 M. Sasani Ghamsari, S. Rahim, S. Radiman and J. Hasani, *Mater. Lett.*, 2012, **85**, 81–83.

8 P. Hernández-Hipólito, N. Juárez-Flores, E. Martínez-Klimova, A. Gómez-Cortés, X. Bokhimi, L. Escobar-Alarcón and T. E. Klimova, *Catal. Today*, 2015, **250**, 187–196.

9 F. L. R. Silva and A. Righi, *Vib. Spectrosc.*, 2017, **88**, 77–82.

10 M. A. Escobedo Bretado, M. A. González Lozano, V. Collins Martínez, A. López Ortiz, M. Meléndez Zaragoza, R. H. Lara and C. U. Moreno Medina, *Int. J. Hydrogen Energy*, 2019, 12470–12476.

11 M. Esmat, A. A. Farghali, S. I. El-Dek, M. H. Khedr, Y. Yamauchi, Y. Bando, N. Fukata and Y. Ide, *Inorg. Chem.*, 2019, **58**, 7989–7996.
